# Age-Related Cognitive Decline and the Olfactory Identification Deficit Are Associated to Increased Level of Depression

**DOI:** 10.3389/fnins.2021.599593

**Published:** 2021-02-22

**Authors:** Fabrizio Sanna, Francesco Loy, Raffaella Piras, Alan Moat, Carla Masala

**Affiliations:** ^1^Section of Neuroscience and Clinical Pharmacology, Department of Biomedical Sciences, University of Cagliari, Monserrato, Italy; ^2^Section of Cytomorphology, Department of Biomedical Sciences, University of Cagliari, Monserrato, Italy; ^3^Section of Physiology, Department of Biomedical Sciences, University of Cagliari, Monserrato, Italy; ^4^Medical Faculty, University of Cagliari, Cagliari, Italy

**Keywords:** smell, taste, depression, age, gender differences, olfaction, cognitive function

## Abstract

**Purpose:**

Previous studies reported a correlation between olfactory function and depression. However, in literature, no data are available for the correlation between depression and all other factors such as age, sex, olfactory, gustatory, and cognitive function in healthy subjects taken together. The aim of this study was to provide a systematic account regarding the association between those variables in a non-clinical population.

**Methods:**

Two hundred and seventy-three participants were recruited with an age range of 19–84 years. Olfactory, gustatory, cognitive function, and depression level were evaluated by means of the following tests: the Sniffin’ Sticks test, Taste Strips test, Montreal Cognitive Assessment (MoCA), and Beck Depression Inventory (BDI).

**Results:**

In our data, an age-related decrease in olfactory and gustatory function and a decline in cognitive functions such as attention, memory, and language were observed. Instead, no significant differences were observed for the depression level in relation to the different age ranges. However, our results indicated that the depression level could be associated to sex, odor identification impairment, and decreased attention and language.

**Conclusion:**

Sex, the odor identification impairment, and an age-related decrease in attention and language are associated with increased level of depression in healthy subjects. Our data can be useful and informative for health care workers, that is, to have adequate preventive strategies to be used whenever these conditions are detected and recognized.

## Introduction

Olfactory and gustatory function allows animals to detect smells and to locate food sources (Loy et al., 2016). In humans, olfactory function regulates food intake, emotional/motivational responses such as pleasantness and reward, anxiety, and social and reproductive behavior (Hoskison, 2013; Brai and Alberi, 2018). The olfactory function usually decreases in relation to age (Doty and Cameron, 2009; Doty and Kamath, 2014; Masala et al., 2018a) due to different age-related events such as a reduction in the expression of olfactory receptors, a decrease in neurogenic processes, an increase in the exposure to toxic environmental agents, and an increase in the ossification and/or closure of foramina on the cribriform plate (Kalmey et al., 1998). Age-related decline in olfactory function could also be associated to a decreased volume of the olfactory bulb (Buschhuter et al., 2008).

Patients with olfactory disorders show usually daily life problems in personal hygiene, safety, sexual behavior, and, particularly food intake (Croy et al., 2014a). Consequently, due to the existence of direct brain connections between olfactory bulb and limbic areas such as the amygdala and orbitofrontal cortex, patients with olfactory dysfunctions exhibited higher levels of depression (Pause et al., 2001; Croy et al., 2010, 2014a, b, c; Negoias et al., 2010). Moreover, patients with depression showed reduced activation in the orbitofrontal cortex, thalamus, and insula (Croy et al., 2014a, b, c) and also a decreased volume of the olfactory bulb (Negoias et al., 2010).

Depression is considered a common disease that affects about 8–12% worldwide people at least once in their life (Andrade et al., 2003). According to the Diagnostic and Statistical Manual of Mental Disorders (American Psychiatric Association [APA], 2013), depression is characterized by different symptoms: depressed mood, a decreased interest or pleasure in all things, weight gain or weight loss, fatigue, insomnia, and/or hypersomnia. Although gustatory disorders are less common among general population than olfactory ones, a connection between taste disorders and depression is also known (Hur et al., 2018); in fact, a previous study reported that alterations in taste and appetite are associated to affective disorders (Maheswaran et al., 2014). Recently, Perini et al. (2019) reported an association between cognitive disorders and depression. However, in literature, these associations with depression were studied independently in patients and no data are available for the correlation between depression and all other factors such as age and sex, and olfactory, gustatory, and cognitive function at the same time in healthy subjects.

The aim of this study was to provide a systematic account regarding the association between the effect of demographic factors (sex and age) and olfactory, gustatory, and cognitive function on the depression level in a non-clinical population.

## Materials and Methods

### Participants

Two hundred and seventy-three participants were recruited (177 females, mean age ± SD, 40.0 ± 18.9 years, and 96 males, mean age ± SD, 41.7 ± 18.5 years, age range 19–84). Among women, no one was pregnant, 57 (32.2%) showed menopause, 120 (67.8%) showed different phases of the menstrual cycle, and 21% (*N* = 37) of them used hormonal contraceptives.

In order to assess statistical differences in olfactory, gustatory, and cognitive function in relation to age, all subjects were divided into three age groups: 18–35 (*N* = 137), 36–55 (*N* = 63), and > 55 (*N* = 73) according to previous studies (Hummel et al., 2007; Masala et al., 2018a, b). Data collection started from September 2018 to December 2019. Exclusion criteria were a history of head or neck trauma, nasal pathology, neurodegenerative diseases, acute respiratory infections, diabetes, stroke, any systemic disease associated with smell disorders, chronic renal disease, and thyroid disorders.

Participants were instructed to drink only water 1 h prior to the experiment and not wear any scented products on the day of testing. Participants received an explanatory statement and gave their written informed consent to participate in the study (Prot. NP/2018/1630).

The clinical evaluation for each participant included age, sex, weight (kg), height (cm), body mass index (BMI), current medications, smoking history, and employment. Moreover, for each subject, olfactory, gustatory, and cognitive function and depression level were assessed.

### Evaluation of the Olfactory Function

Olfactory function was evaluated using the Sniffin’ Sticks test (Burghart Messtechnik, Wedel, Germany), taking into consideration three different parameters: odor threshold (OT), odor discrimination (OD), and odor identification (OI) (Hummel et al., 1997, 2007; Oleszkiewicz et al., 2019). First, OT was determined using n-butanol with 16 stepwise dilutions. Thresholds were measured using the single-staircase technique based on a three-alternative forced-choice task (3AFC). Second, OD was measured over 16 trials (Masala et al., 2018a, b; Masala et al., 2019; Masala et al., 2020; Solla et al., 2020). For each discrimination, three pens were presented, two containing the same odor and the third containing the target odorant (3AFC task). Third, OI was measured using 16 common odors, each presented with four verbal descriptors in a multiple forced-choice format (three distractors and one target). The interval between odor presentations was 20–30 s. A total score (Threshold, Discrimination, Identification, TDI) was calculated: a score of > 30.5, between 30.5 and 16.5, and < 16.5 indicated normosmia, hyposmia, and functional anosmia, respectively (Hummel et al., 2007).

### Assessment of Gustatory Function

Taste function was examined by the Taste Strips test (TST) (Mueller et al., 2003; Hummel et al., 2007; Landis et al., 2009). TST is a validated method for the evaluation of taste sensitivity using 16 spoon-shaped filter strips impregnated with four concentrations of the four basic tastes (sweet, sour, salty, and bitter). The TST strips were positioned on the tongue, and the subject was asked to identify the taste quality from a list of four descriptors (sweet, sour, salty, and bitter) in a forced-choice paradigm. Before each strip administration, the mouth was cleaned with water. The TST global score is the sum of correct identifications and defines the taste performance. A TST score ≥ 9 is considered as normogeusia and a TST score < 9 is classified as hypogeusia.

### Assessment of Cognitive Function

A cognitive function evaluation was carried out using the Montreal Cognitive Assessment (MoCA), which assesses cognitive impairment in eight different domains: visual–constructional skills, executive functions, attention and concentration, memory, verbal fluency (language), conceptual thinking, calculations, and spatial orientation (Conti et al., 2015; Cecchini et al., 2019).

### Depression Level Assessment

Depression level was evaluated using the self-reported Beck Depression Inventory (BDI-II) test (Beck et al., 1961), which includes 21 items with a four-point scale ranging in order of severity from 0 to 3. The depression level was classified as minimal = 0–13, mild depression = 14–19, moderate depression = 20–28, and severe depression = 29–63.

### Statistics

At first, sample size calculation was performed in order to assess the required minimum number of subjects to be enrolled in the study. Based on previous studies using similar protocols (Masala et al., 2018a, b; Cecchini et al., 2019), a number of about 200–250 total subjects were considered adequate in order to detect the investigated differences. In fact, a power calculation, based on similar studies and considering a critical effect size *f* = 0.25 (medium effect), with 85–90% power and a 5% significance level in a standard one-way ANOVA, suggested a minimal required number of about 200 total subjects, and a power calculation considering a critical effect size *d* = 0.5 (medium effect), with 85–90% power, and a two-tailed 5% significance level in an unpaired *t* test, suggested a required minimal number of 70/80 subjects/group.

Data were presented as mean values ± standard deviation (SD). In particular, between-subjects one-way ANOVAs and *post hoc* analyses using multiple pairwise comparison tests with Bonferroni’s corrected alpha values were carried out to assess statistical differences in demographic information (weight, height, and BMI); olfactory, gustatory, and cognitive function; and depression level in the three different age ranges (18–35, 36–55, and > 55 years).

In addition, an effect size estimation (Cohen’s *d* for any significant pairwise comparison) was also reported in the *Results* section where appropriate (a value of 0.2, 0.5, or 0.8 indicates a small, medium, or large effect size, respectively). Differences in the frequencies of the three age groups for the depression level were assessed using the chi-square (χ^2^) test. In order to identify the more promising factors for the multivariate regression analyses, bivariate correlations between depression vs. sex, age, weight, BMI, and olfactory, gustatory, and cognitive functions were assessed using Pearson’s coefficient (*r*).

Furthermore, an exploratory stepwise multivariate linear regression analysis was performed to assess the potential contribution of each significant correlated factor (such as age, sex, OI deficit, impairment in language, and attention) on the depression level. In the multivariate linear regression analysis, the depression level (BDI score) was set as the dependent variable, while age, sex, OI, attention, and language were independent variables (predictors).

In order to perform the multivariate linear regression analysis using a stepwise selection, in model 1, we calculated the correlation between depression level with two independent variables, such as age and sex, then in model 2, we included the OI, and, finally, in model 3, we added attention and language. This stepwise method allows us to evaluate the specific role of each independent variable in the model.

Statistical analyses were performed by the SPSS software version 22 for Windows (IBM, Armonk, NY, United States). The significance level was set at *p* < 0.05.

## Results

Mean values ± SD of weight, height, and BMI in the three age groups were reported in Table 1. Results showed a significant difference in weight [*F*_(__2,270__)_ = 6.782, *p* < 0.001, partial η^2^ = 0.048] and in BMI [*F*_(__2,270__)_ = 22.346, *p* < 0.01, partial η^2^ = 0.142]; in particular, an increase in both weight and BMI in elderly subjects (>55 years) compared to younger participants (18–35 years) (both *p* < 0.01, Bonferroni’s *post hoc* multiple comparison test; *d* = 0.52 and *d* = 0.91, for weight and BMI, respectively). Conversely, no significant differences were observed in the height between the three age groups.

**TABLE 1:** Weight, height, and BMI of participants (mean ± SD).

	16–35 years	36–55 years	>55 years	General ANOVA	Bonferroni’s multiple comparisons test
Weight (kg)	61.3 ± 12.707	64.4 ± 12.706	68.5 ± 15.023	***p* < 0.001**	*p* < 0.01*
Height (cm)	163 ± 19.528	167 ± 9.197	160 ± 19.616	*p* > 0.05	*p* > 0.05
BMI	22.2 ± 4.189	23 ± 3.129	26.1 ± 4.421	***p* < 0.01**	*p* < 0.01*

Bold indicates a significant level. *Statistical differences between younger (16–35 years) and elderly participants (>55 years). BMI, body mass index.

As regards the olfactory function, mean values ± SD for OT were 7.4 ± 4.416, 5.5 ± 2.577, and 5.4 ± 3.795 in the three age groups, respectively (Table 2). A significant difference in OT [*F*_(__2,270__)_ = 8.538, *p* < 0.001, partial η^2^ = 0.059] was found, particularly an impairment in both the middle age (36–55 years) and the elderly (> 55 years) compared to the younger subjects (18–35 years) (*p* < 0.01 and *p* < 0.001, *d* = 0.53 and *d* = 0.49, respectively). Mean values ± SD were 12.4 ± 1.696, 12.1 ± 1.748, and 10.8 ± 2.436 for OD and 13.3 ± 1.535, 13.5 ± 1.480, and 12.1 ± 2.463 for OI, in the three age groups, respectively (Table 2). In the OD test, a significant performance decrease [*F*_(__2,270__)_ = 15.852, *p* < 0.001, partial η^2^ = 0.105] was observed, in particular in elderly (age > 55 years) compared to younger (18–35 years) (*p* < 0.001; *d* = 0.77) and middle age (36–55 years) subjects (*p* < 0.01; *d* = 0.63). Statistical differences [*F*_(__2,270__)_ = 12.122, *p* < 0.01, partial η^2^ = 0.082] were also found in the OI between both younger (16–35 years) and middle age (36–55 years) vs. elderly participants (>55 years) (both *p* < 0.01; *d* = 0.57 and *d* = 0.68, respectively). Consequently, a significant impairment in TDI score [*F*_(__2,270__)_ = 20.623, *p* < 0.001, partial η^2^ = 0.133] was found, in particular, in the elderly (>55 years) compared to both the younger (18–35 years) (*p* < 0.001; *d* = 0.83) and middle age subjects (36–55 years) (*p* < 0.01; *d* = 0.56). Mean values ± SD for the TDI score were 32.7 ± 4.230, 31.1 ± 3.514, and 28.3 ± 6.243 in the three age groups, respectively (Table 2).

**TABLE 2:** Statistical differences for olfactory function in the three age groups (mean ± SD).

Sniffin’ sticks	16–35 years	36–55 years	>55 years	General ANOVA	Bonferroni’s multiple comparisons test
OT	7.4 ± 4.416	5.5 ± 2.577	5.4 ± 3.795	***p* < 0.001**	*p* < 0.001* *p* < 0.01^#^
OD	12.4 ± 1.696	12.1 ± 1.748	10.8 ± 2.436	***p* < 0.001**	*p* < 0.001* *p* < 0.01^+^
OI	13.3 ± 1.535	13.5 ± 1.480	12.1 ± 2.463	***p* < 0.01**	*p* < 0.01* *p* < 0.01^+^
TDI	32.7 ± 4.230	31.1 ± 3.514	28.3 ± 6.243	***p* < 0.001**	*p* < 0.001* *p* < 0.01^+^

OD, Odor discrimination; OI, odor identification; OT, odor threshold; TDI, total score. Bold indicates a significant level. *Statistical differences between younger (16–35 years) and elderly participants (>55 years). ^#^ Statistical differences between middle age (36–55 years) and younger participants (16–35 years). ^+^Statistical differences between middle age (36–55 years) and elderly subjects (>55 years).

As regards gustatory function, significant differences among the three age groups were found only for the salty taste [*F*_(__2,270__)_ = 3.037, *p* < 0.05, partial η^2^ = 0.022] and the total taste score [*F*_(__2,270__)_ = 27.731, *p* < 0.01, partial η^2^ = 0.133] (Table 3). In particular, elderly subjects (>55 years) showed a significant decrease in salty perception and in total taste performance compared to younger participants (18–35 years) (both *p* < 0.05; *d* = 0.38 and *d* = 0.46, respectively). Conversely, no significant differences among the three age groups were found for sweet, sour, and bitter taste (*p* > 0.05).

**TABLE 3:** Statistical differences for gustatory function in the three age groups (mean ± SD).

Taste Strip test	16–35 years	36–55 years	>55 years	General ANOVA	Bonferroni’s multiple comparisons test
Sweet	3.3 ± 0.596	3.1 ± 0.942	3.1 ± 0.811	*p* > 0.05	*p* > 0.05
Salty	3.2 ± 0.923	3 ± 1.062	2.8 ± 1.232	***p* < 0.05**	*p* < 0.05*
Sour	2.5 ± 0.984	2.4 ± 0.962	2.2 ± 1.003	*p* > 0.05	*p* > 0.05
Bitter	3.1 ± 1.114	3 ± 1.084	2.8 ± 1.367	*p* > 0.05	*p* > 0.05
Total taste score	12.1 ± 2.012	11.6 ± 2.588	11 ± 2.731	***p* < 0.05**	*p* < 0.05*

Bold indicates a significant level. *Statistical differences between younger (16–35 years) and elderly participants (>55 years).

As regards cognitive functions, mean values ± SD assessed by the MoCA test were indicated in Table 4. Mean values ± SD for memory performance were 3.7 ± 1.311, 2.8 ± 1.588, and 1.9 ± 1.545 in the three age groups, respectively (Table 4), and a significant age-related decrease was observed [*F*_(__2_,_270__)_ = 35.314, *p* < 0.001, partial η^2^ = 0.207]. Statistical differences in memory scores were observed between younger (16–35 years), middle age (36–55 years), and elderly participants (>55 years) (both *p* < 0.001; *d* = 1.28 and *d* = 0.58, respectively). Furthermore, mean values ± SD for attention were 5.9 ± 0.296, 5.4 ± 0.752, and 5.2 ± 1.100 in the three age groups, respectively. A significant decrease in attention [*F*_(__2,270__)_ = 19.706, *p* < 0.01, partial η^2^ = 0.127] was found, in particular, in the elderly participants (>55 years) compared to the younger ones (18–35 years) (*p* < 0.01; *d* = 0.87), while no significant differences were observed between middle aged (36–55 years) and elderly participants (>55 years). For language, mean values ± SD were 2.6 ± 0.296, 2.7 ± 0.496, and 2.1 ± 0.812 in the three age groups, respectively (Table 4). Also, in this case, significant age-related differences were detected [*F*_(__2,270__)_ = 20.596, *p* < 0.01, partial η^2^ = 0.132], with elderly subjects (>55 years) showing a significant decrease in language function compared to the younger ones (18–35 years) (*p* < 0.01; *d* = 0.83). Conversely, no significant differences were observed in naming, abstraction, orientation, and spatial executive functions among the three age groups (all *p* > 0.05). Finally, according to the differences observed in memory, attention, and language MoCA subtests, differences were also detected in the total MoCA score [*F*_(__2,270__)_ = 33.944, *p* < 0.001, partial η^2^ = 0.201], with elderly subjects (>55 years) showing a significant impairment in total cognitive functions compared to the younger ones (*p* < 0.001; *d* = 1.04). In particular, mean values ± SD for total cognitive functions (MoCA) were 27.8 ± 1.745, 26.8 ± 2.179, and 24.8 ± 3.675 in the three age groups, respectively (Table 4).

**TABLE 4:** Statistical differences for cognitive abilities (MoCA) in the three age groups (mean ± SD).

	16–35 years	36–55 years	>55 years	General ANOVA	Bonferroni’s multiple comparisons test
Abstraction	1.9 ± 0.459	1.9 ± 0.246	1.8 ± 0.420	*p* > 0.05	*p* > 0.05
Attention	5.9 ± 0.296	5.4 ± 0.752	5.2 ± 1.100	***p* < 0.01**	*p* < 0.01*
Language	2.6 ± 0.296	2.7 ± 0.496	2.1 ± 0.812	***p* < 0.01**	*p* < 0.01*
Memory	3.7 ± 1.311	2.8 ± 1.588	1.9 ± 1.545	***p* < 0.001**	*p* < 0.001***
Naming	3 ± 0.01	3 ± 0.215	3 ± 0.379	*p* > 0.05	*p* > 0.05
Orientation	6 ± 0.378	6 ± 0.126	6 ± 0.01	*p* > 0.05	*p* > 0.05
Spatial executive	4.9 ± 0.421	4.9 ± 0.458	4.9 ± 1.166	*p* > 0.05	*p* > 0.05
Cognitive ability (MoCA)	27.8 ± 1.745	26.8 ± 2.179	24.8 ± 3.675	***p* < 0.001**	*p* < 0.001*

Bold indicates a significant level. *Statistical differences between younger (16–35 years) and elderly participants (>55 years). ***Statistical differences between younger (16–35 years), middle age (36–55 years), and elderly participants (>55 years).

As regards the depression level, no significant differences were observed in the distribution of BDI scores among the three age groups; however, a non-significant trend (chi-square test, χ^2^ = 2.443, *p* = 0.059) was observed in the comparison between younger and elderly subjects for minimal vs. mild/moderate depression level (Table 5). In particular, among the younger group (18–35 years), 87.5% of the participants exhibited minimal depression, 9.5% showed mild depression, and only 2.9% had moderate depression (Table 5), while among the 36–55-year group, 84% of the subjects showed minimal depression, 11% exhibited mild depression, and 5% showed moderate depression. Instead, in the elderly group (>55 years), 79.4% of the participants exhibited minimal depression, 15% showed mild depression, and 5.5% exhibited moderate depression. Not one of the enrolled participants showed a severe level of depression.

**TABLE 5:** Depression levels (mean ± SD) assessed using the Beck Depression Inventory (BDI) test and their distribution among the three age groups.

BDI scores	18–35 years	36–55 years	>55 years
BDI total score (mean ± SD)	6.7 ± 5.248	7.3 ± 6.907	7.5 ± 7.640
0–13 (minimal depression)	120 (87.5%)	53 (84%)	58 (79.4%)
14–19 (mild depression)	13 (9.5%)	7 (11%)	11 (15%)
20–28 (moderate depression)	4 (2.9%)	3 (5%)	4 (5.5%)

In addition, in order to evaluate all correlations between depression level and each other factor (sex, age, BMI, and olfactory, gustatory, and cognitive functions), we performed bivariate correlations. Significant negative correlations were found between the depression level vs. sex (*r* = −0.221, *p* < 0.01), OI (*r* = −0.115, *p* < 0.05), attention (*r* = −0.196, *p* < 0.01), and language (*r* = −0.180, *p* < 0.01). Conversely, no significant correlations between the depression level vs. weight, BMI, memory, spatial/executive function, orientation, abstraction, naming, OT, OD, and gustatory function were observed (Table 6).

**TABLE 6:** Pearson’s correlations.

Factor	Pearson’s correlation (*r*)	Significance (*p*–value)
Depression	1.000	−
Sex	**−0.221**	**>0.01**
Age	–0.015	0.404
Weight	–0.028	0.324
BMI	0.094	0.060
OT	0.013	0.417
OD	–0.031	0.305
OI	**−0.115**	**0.029**
Sweet taste	–0.019	0.378
Salty taste	0.019	0.375
Sour taste	0.024	0.349
Bitter taste	–0.009	0.439
Spatial executive	–0.040	0.253
Naming	–0.007	0.454
Memory	–0.086	0.078
Attention	**−0.196**	**>0.01**
Language	**−0.180**	**>0.01**
Abstraction	–0.058	0.171
Orientation	0.020	0.373

*Bold indicates a significant level. BMI, Body mass index; OD, Odor discrimination; OI, odor identification; OT, odor threshold.*

Finally, in order to investigate the contribution of sex, age, OI, attention, and language on the depression level, an exploratory stepwise multivariate linear regression analysis was performed. The depression level was considered as the dependent variable, while in model 1, sex and age were the independent variables (predictors). A significant contribution emerged [*F*_(__2,270__)_ = 6.948, *p* < 0.001] and the model explained around 5% of variance (*R*^2^ = 0.049). Instead, in model 2, a significant effect was observed for sex, age, and OI [*F*_(__2,269__)_ = 6.238, *p* ≤ 0.001] with an explanation of around 7% of variance (*R*^2^ = 0.065). Finally, in model 3, a significant contribution was observed for sex, age, OI, attention, and language [*F*_(__2,267__)_ = 6.564, *p* ≤ 0.001]. Model 3 explained around 11% of variance (*R*^2^ = 0.109) (Table 7).

**TABLE 7:** Multiple linear regression analyses.

Predictors	*B*	SD Error	Beta	*t*	Significance (*p-*value)
**Depression (dependent variable)**					
**Model 1**					
Sex	–2.756	0.741	–0.221	–3.719	**>0.001**
Age	0.001	0.019	0.002	0.041	0.968
**Model 2**					
Sex	–2.777	0.736	–0.223	–3.774	**>0.001**
Age	–0.011	0.019	–0.033	–0.543	0.588
OI	–0.415	0.193	–0.132	–2.152	**0.032**
**Model 3**					
Sex	–2.252	0.735	–0.181	–3.062	**0.002**
Age	–0.041	0.021	–0.130	–1.971	0.053
OI	–0.304	0.192	–0.097	–1.584	0.114
Attention	–1.219	0.501	–0.160	–2.434	**0.016**
Language	–1.250	0.560	–0.138	–2.230	**0.027**

*OI, odor identification. Bold indicates a significant level.*

## Discussion

This study evaluated for the first time the association between depression and age-related decline in olfactory, gustatory, and cognitive function in healthy subjects. In general, our results indicated that depression could be associated to sex, OI impairment, and decreased attention and language. In line with previous studies (Altemus, 2006; Labaka et al., 2018), our data showed higher depression scores in women compared to men. A possible explanation for this gender-related difference in depression may be linked to the different levels in inflammatory cytokines (Felgel and Lotrich, 2013; Slavich and Sacher, 2019), to neurotrophic and hormonal factors (Kuehner, 2017), as well as to differences in central serotoninergic function (Gressier et al., 2016; Perry et al., 2017). Moreover, as reported in several studies (see, for example, Altemus, 2006, and references therein), women show greater influence of reproductive hormones across their life such as changes in reproductive hormones during puberty, the menstrual cycle, pregnancy, and menopause, which may alter brain function and increase the susceptibility to develop depressive conditions. In particular, hormonally induced changes in the brain levels of norepinephrine, oxytocin, prolactin, and GABA during pregnancy and lactation may induce depression (Altemus, 2006).

As regards age-related depression, our data showed no significant differences in depression level mean values among subjects in the three age ranges. A possible explanation for these data can be linked to the fact that around 80% of subjects in our sample showed depression scores between 0 and 13, so all participants showed a minimal depression level. In contrast, a previous study (Goldberg et al., 2003) reported mean depression values in elderly patients that ranged between 10 and 13. However, differences between this study and our data could be associated to general differences in sample characteristics (healthy subjects vs. medical outpatients of a behavioral medicine clinic), the diverse inclusion/exclusion criteria, or genetic, individual (such as emotional), and social (such as cultural) characteristics of participants (Laird et al., 2019).

Moreover, in line with a previous study (Starkstein and Kremer, 2001), our results showed a correlation between depression level vs. decreased attention and language. This aspect could be explained considering an age-related decrease in memory, attention, language, and total cognitive performance in elderly subjects (with >55 years). Usually, the diagnosis for age-related cognitive decline requires a reliable documentation of cognitive impairment in two or more cognitive domains such as memory, attention, and language. This deficit is classified as “age-related cognitive decline” in the DSM-IV, Diagnostic and Statistical Manual of Mental Disorders, fourth edition (American Psychiatric Association [APA], 2000), while in the DSM-V (American Psychiatric Association [APA], 2013), it is indicated as a “neurocognitive disorder.” This age-related cognitive decline could be associated to a decrease in brain volume such as a degeneration in fronto-temporal, parietal, and occipital lobes (Peters, 2006). Moreover, the brain of elderly subjects, for at least some of these, may also show similar initial alterations as those observed in Alzheimer’s disease such as the presence of neurofibrillary tangles and senile plaques (Serrano-Pozo et al., 2011).

In addition, our results showed a significant negative association between depression and OI, while no associations were found for OT and OD. This negative correlation, as previously indicated by Zucco and Bollini (2011), could suggest a possible association between olfactory deficit in depression and impairment in cognitive function and in memory.

Our data are in line with previous studies reporting a significant association between depression and olfactory dysfunction (Atanasova et al., 2008; Croy et al., 2014a, c). This association may be explained considering the neuro-anatomical connections between olfactory, emotional, and cognitive brain areas as previously reported by Soudry et al. (2011), in view of the fact that OI is usually associated to cognitive and emotional central pathways connecting the orbitofrontal cortex, piriform cortex, hippocampal and entorhinal cortex, and the amygdala, while OT is more associated to individual differences of the nasal cavity (Masala et al., 2019). Previous studies (Doty et al., 1987; Tabert et al., 2005) indicated that the most common impairment in elderly adults without cognitive impairment is the OI deficit, which consists in the identification of a specific odor at supra-threshold concentration. Conversely, deficit in OD occurs in patients with mild cognitive impairment, or in subjects with Alzheimer’s disease and with REM sleep behavior disorder (Doty et al., 1987; Tabert et al., 2005; Galbiati et al., 2019).

In our sample, no significant associations were found between depression vs. weight and BMI. However, our results showed that elderly subjects (with >55 years) exhibited an increase in weight and BMI compared to young participants (18–35 years). These results are in line with previous studies (Reas et al., 2007; Flegal et al., 2013; Yi et al., 2015) that reported an increased prevalence of obesity in elderly subjects. A possible explanation for this result could be related to an age-related decrease in olfactory and gustatory function. As indicated in previous studies (Hummel et al., 2001; Pribitkin et al., 2003; Gudziol et al., 2007; Landis et al., 2010; Stinton et al., 2010; Boesveldt et al., 2011), patients with olfactory disorders also showed a decreased gustatory function, and this suggests the presence of mutual interactions among olfactory and gustatory senses. Different theories were reported in order to explain this age-related decrease in olfactory function such as a decrease in the number of fibers in the olfactory bulb and a reduction in the number of olfactory receptor neurons (Boyce and Shone, 2006). The age-related decline in olfactory function could also be associated to a decreased volume of the olfactory bulb (Buschhuter et al., 2008). Instead, the age-related decrease in gustatory function could be related to a reduction of the fungiform taste papillae density and to changes in salivary composition (Walliczek-Dworschak and Hummel, 2017).

## Conclusion

Our data, in line with previous studies, confirm a decrease in olfactory function, an impairment in perception of salty, and a decline in memory, attention, and language related to the age. Conversely, no significant differences were observed for the depression level in relation to the different age groups. However, OI impairment and decrease in attention and language are associated with increased depression levels.

Depression is a multifactorial broadly diffused psychopathology that increases all over the world. Also, in consideration of the potential harmful consequences in people affected by depression (such as a significant negative burden in quality of life, high rates of relapse, and suicide risk), an early detection of depression becomes a factor of primary importance in the population. Our data can be useful and informative for health care workers in order to have adequate preventive strategies when depressive conditions are detected and recognized.

## Data Availability Statement

The raw data supporting the conclusions of this article will be made available by the authors under request.

## Ethics Statement

The studies involving human participants were reviewed and approved by the Comitato etico Azienda Ospedaliero Universitaria di Cagliari (PROT. NP/2018/1630). The patients/participants provided their written informed consent to participate in this study.

## Author Contributions

CM and FS: conceptualization, supervision, formal analysis. CM, FL, and RP: data curation. CM: funding acquisition, project administration, validation. FL and RP: investigation, methodology. FL: software, visualization. RP and CM: writing—original draft. FS and AM: writing—review and editing. All authors have read and agreed to the published version of the manuscript.

## Conflict of Interest

The authors declare that the research was conducted in the absence of any commercial or financial relationships that could be construed as a potential conflict of interest.
